# Supplementary material for: The Antidiabetic Agent Metformin Inhibits IL-23 Production in Murine Bone-Marrow-Derived Dendritic Cells
Source: J Clin Med. 2021 Nov 29;10(23):5610. doi: 10.3390/jcm10235610 (PMC8658299; doi:10.3390/jcm10235610)
Supplement: Supplementary file 1 [file jcm-10-05610-s001.zip › jcm-1456400-supplementary.pdf]

Table S1. Murine TaqMan gene expression assay probes.

Primer	Assay ID
Ywhaz	Mm03950126-s1
IL-23	Mm00518984-m1
Nfkbiz	Mm00600522-m1
